# What do people really think of generic medicines? A systematic review and critical appraisal of literature on stakeholder perceptions of generic drugs

**DOI:** 10.1186/s12916-015-0415-3

**Published:** 2015-07-29

**Authors:** Suzanne S. Dunne, Colum P. Dunne

**Affiliations:** Centre for Interventions in Infection, Inflammation and Immunity (4i), Graduate Entry Medical School, University of Limerick, Limerick, Ireland

**Keywords:** Generic medicine, Generic drug, Systematic review, Perceptions, Opinions, Stakeholders, Patient, Physician, Pharmacist

## Abstract

**Background:**

Considerable emphasis is presently being placed on usage of generic medicines by governments focussed on the potential economic benefits associated with their use. Concurrently, there is increasing discussion in the lay media of perceived doubts regarding the quality and equivalence of generic medicines. The objective of this paper is to report the outcomes of a systematic search for peer-reviewed, published studies that focus on physician, pharmacist and patient/consumer perspectives of generic medicines.

**Methods:**

Literature published between January 2003 and November 2014, which is indexed in PubMed and Scopus, on the topic of opinions of physicians, pharmacists and patients with respect to generic medicines was searched, and articles within the scope of this review were appraised. Search keywords used included perception, opinion, attitude and view, along with keywords specific to each cohort.

**Results:**

Following review of titles and abstracts to identify publications relevant to the scope, 16 papers on physician opinions, 11 papers on pharmacist opinions and 31 papers on patient/consumer opinions were included in this review. Quantitative studies (*n* = 37) were the most common approach adopted by researchers, generally in the form of self-administered questionnaires/surveys. Qualitative methodologies (*n* = 15) were also reported, albeit in fewer cases. In all three cohorts, opinions of generic medicines have improved but some mistrust remains, most particularly in the patient group where there appears to be a strongly held belief that less expensive equals lower quality. Acceptance of generics appears to be higher in consumers with higher levels of education while patients from lower socioeconomic demographic groups, hence generally having lower levels of education, tend to have greater mistrust of generics.

**Conclusions:**

A key factor in improving confidence in generic products is the provision of information and education, particularly in the areas of equivalency, regulation and dispelling myths about generic medicines (such as the belief that they are counterfeits). Further, as patient trust in their physician often overrules their personal mistrust of generic medicines, enhancing the opinions of physicians regarding generics may have particular importance in strategies to promote usage and acceptance of generic medicines in the future.

## Background

Generic medicines are those where the original patent has expired and which may now be produced by manufacturers other than the original innovator (patent-holding) company. The term ‘generic drug’ or ‘generic medicine’ is commonly understood, as defined by the World Health Organization (WHO), to mean a pharmaceutical product that is usually intended to be interchangeable with an innovator product, is manufactured without a licence from the innovator company and is marketed after the expiry date of the patent or other exclusive rights [1]. Other definitions of generic medicines may be subtly different, for example the US Food and Drug Administration (FDA) defines a generic as: ‘A drug product that is comparable to a brand/reference listed drug product in dosage form, strength, route of administration, quality and performance characteristics, and intended use’ [2]; and the European Medicines Agency (EMA) definition is: ‘A generic medicine is a medicine that is developed to be the same as a medicine that has already been authorised (the ‘reference medicine’). A generic medicine contains the same active substance(s) as the reference medicine, and it is used at the same dose(s) to treat the same disease(s) as the reference medicine. However, the name of the medicine, its appearance (such as colour or shape) and its packaging can be different from those of the reference medicine’ [3]. However, all agree on the general requirements that the product is off-patent, contains an active ingredient in a previously approved medicine, is shown to be bioequivalent to that previously approved medicine, and has the same dosage form, route of administration and treatment characteristics.

Global healthcare expenditure is increasing steadily [4] and generic medicine utilisation is often encouraged as a cost-containment measure [5], as generic medicines are generally, but not always, less expensive than their proprietary counterparts; the manifold reasons for which (including the lack of necessity to recoup research and development costs, payer pressures and market competition) are discussed in a recent review article [6]. As generic medicines can be priced as low as 2–10 % of pre-patent loss prices, their use can lead to considerable savings [7, 8]. As a result, emphasis is being placed on usage of generic medicines by governments focussed on the potential economic benefits and multiple strategies have been introduced across countries to enhance their use [9]. Consequently, there is increasing discussion in the lay media of perceived doubts regarding the quality and equivalence of generic medicines. In parallel, there have been a number of studies completed in diverse territories that have assessed opinions, knowledge, attitudes and awareness of generic medicines amongst both healthcare professionals and members of the general public. However, while there have been review papers that have reported the views of these individual cohorts, there has not, to date, been a single comprehensive review evaluating and critically appraising, in one easily accessible paper, the collective literature (that is, collating views of medicine prescribers, dispensers and consumers together) in this area. Hence, one of the objectives of this report is to collate the views of each of the key stakeholder groups in medicine provision, those being physicians, pharmacists and patients/consumers.

While many countries have introduced substitution of prescribed branded medications with less expensive generic equivalents, as well as other measures to increase the prescribing and dispensing of generic medicines [10–12], it has been reported that negative opinions held about generic medicines (which can be generally summarised as the view that generics are less effective and/or of poorer quality than proprietary, branded medicines) – by both professionals [13–16] and members of the general public [17–20] – have the potential to reduce acceptance of generics in healthcare provision.

It is important to note that strict regulations for bioequivalence (that is, a formal demonstration of equivalence between the generic and the proprietary formulations of the drug) exist; for example, in the USA [21] and Europe [22], with studies in the USA having reported that pharmacokinetic properties of generic drugs, as determined by extent of exposure (area under the concentration-time curve (AUC)) and mean peak serum drug concentration (Cmax), differed by only 3–4 % on average from those of the originator [23]. Products manufactured to standards that do not fulfill key criteria for bioequivalence are not granted marketing authorisation as generic medicines. Furthermore, meta-analyses and other studies have shown no difference in outcomes between originator (proprietary) medicines and their generic equivalents across a wide range of medicine types and clinical issues; for example, cardiovascular drugs [24], antipsychotics [25], antiepilepsy medicines [26] and antibiotics [27]. While some controversy remains, as will be discussed later, there have been repeated attempts by proprietary manufacturers to cast doubt on the quality of generic medicines and to hinder entry of generic products into the marketplace; for example, in 2013 the French Competition Authority levied fines of €40.6 million on Sanofi-Aventis for disparaging generic versions of Plavix (clopidogrel) and for discouraging generic substitution of this product [28].

As much debate still surrounds the topic of usage of generic drugs – medicines, which, as mentioned earlier are often vital to attempts to maintain control of healthcare costs – it is important to understand the opinions held by stakeholders in relation to these medicines and their usage. Hence the primary objective of this paper is to report the outcomes of a systematic search for studies that focus on physician, pharmacist and patient opinions (including perspectives, behaviours, attitudes and knowledge, where applicable in different publications) of generic medicines; and to critically appraise their methodologies and findings with a view to providing unambiguous understanding of available literature, in order to inform researchers and policy makers as to gaps in knowledge and the challenges that remain regarding increasing usage of generic medicines. To ensure that all relevant publications were incorporated into this review a variety of keywords related to these topics were used for searches and these included: ‘perception’; ‘view’; ‘opinion’; ‘attitude’; ‘behaviour’; and ‘knowledge’, and these terms have been used throughout this review.

## Methods

### Scope

Literature published between January 2003 and November 2014, which is indexed in PubMed and Scopus, on the topic of opinions of physicians, pharmacists and patients/consumers with respect to generic medicines.

### Systematic approach to finding appropriate literature

Searches were performed in PubMed and Scopus in November 2014 for full articles published on the topic of perceptions/opinions/behaviours/views relating to generic medicines amongst the specific stakeholder groups of physicians/general practitioners, pharmacists and patients. Any study methodology leading to a publication within the scope of this review was included. Papers that were not published in English were excluded. Only full, original research papers and reviews were included. Editorial opinions, letters to the editor and other ‘opinion’-based publications were not included.

#### Search methodology

Title and abstract fields were searched for publications containing the words: generic; perception; opinion; attitude; or view, along with the modifiers for each stakeholder group. For the patient group, both ‘patient’ and ‘consumer’ were searched for; for the pharmacist group, the word ‘pharmacist’ was used; and for the physician group both ‘physician’ and ‘general practitioner’ or ‘GP’ were the terms used. Boolean operators were used to combine search components and truncation was used with the stakeholder search terms, to capture as many search results as possible. MeSH (Medical Subject Headings) terms were made use of where applicable.

For example, the PubMed searches were:

- ((pharmacist*) OR (“pharmacists”[MeSH Terms] OR “pharmacists”[All Fields] OR “pharmacist”[All Fields])) AND (opinion[All Fields] OR (“perception”[MeSH Terms] OR “perception”[All Fields]) OR view[All Fields]) AND ((“drugs, generic”[MeSH Terms] OR (“drugs”[All Fields] AND “generic”[All Fields]) OR “generic drugs”[All Fields] OR “generic”[All Fields])) AND generic AND (perception OR opinion OR attitude OR view)[Title/Abstract]
- (general practitioner* OR physician*) OR ((“general practitioners”[MeSH Terms] OR (“general”[All Fields] AND “practitioners”[All Fields]) OR “general practitioners”[All Fields] OR (“general”[All Fields] AND “practitioner”[All Fields]) OR “general practitioner”[All Fields]) OR (“physicians”[MeSH Terms] OR “physicians”[All Fields] OR “physician”[All Fields])) AND (opinion[All Fields] OR (“perception”[MeSH Terms] OR “perception”[All Fields]) OR view[All Fields]) AND ((“drugs, generic”[MeSH Terms] OR (“drugs”[All Fields] AND “generic”[All Fields]) OR “generic drugs”[All Fields] OR “generic”[All Fields]))
- (patient[Title/Abstract] OR consumer[Title/Abstract]) AND generic[Title/Abstract] AND (perception[Title/Abstract] OR opinion[Title/Abstract] OR attitude[Title/Abstract]) AND generic AND (perception OR opinion OR attitude OR view)[Title/Abstract]
- (patient*[Title/Abstract] OR consumer*[Title/Abstract]) AND generic*[Title/Abstract] OR (perception[Title/Abstract] OR opinion[Title/Abstract] OR attitude[Title/Abstract]) AND (opinion[All Fields] OR (“perception”[MeSH Terms] OR “perception”[All Fields]) OR view[All Fields]) AND ((“drugs, generic”[MeSH Terms] OR (“drugs”[All Fields] AND “generic”[All Fields]) OR “generic drugs”[All Fields] OR “generic”[All Fields])).

#### Critical appraisal and synthesis

While no papers were excluded based on a subjective assessment of the quality of the reports – as per criteria described in the journal *Nature Clinical Practice* [29] (specifically, the recommended criteria include: relevance of the paper to the topic; whether the paper contributes new knowledge; type of research questions being asked; whether the study design is appropriate; has the potential for bias been addressed; was the study completed as per protocol; did the study test a stated hypothesis; was the statistical analysis appropriate; do the data justify the conclusions drawn; and has potential conflict of interest been identified) and the Critical Appraisal Skills Programme (CASP) checklist [30] – emphasis was placed broadly on what the key results were, whether the results were valid and whether the studies were relevant to the topic of stakeholder perceptions of generic medicines.

Data were extracted from the included articles. Patient, physician and pharmacist perspectives were defined as first-order constructs, and the authors’ interpretations of these constructs were defined as second-order constructs.

## Results

**Fig. 1:**
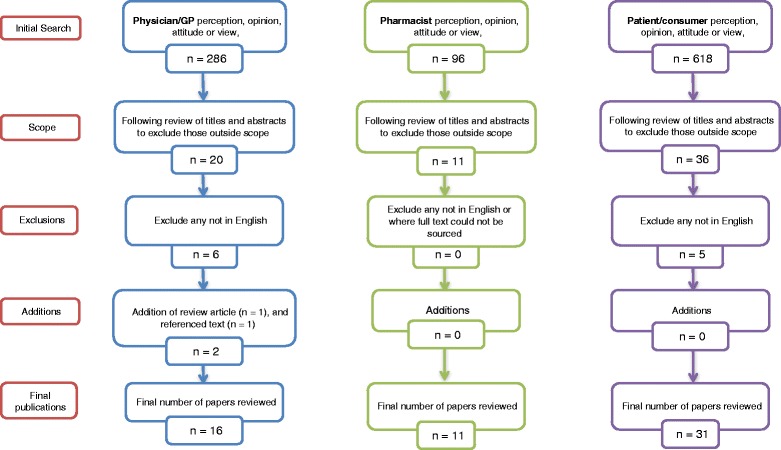
Flowcharts of selection methodology by stakeholder group

The PubMed search returned the greatest number of publications. The physician/general practitioner search returned 286 articles, the patient/consumer search returned 618 articles and the pharmacist search returned 96 articles. The headings and abstracts of those 1,000 publications were reviewed to determine which were within the scope of this review. Figure 1 shows the approach taken and the number of publications obtained. The exclusion of papers was on the basis of reading their titles and abstracts, and the subsequent determination that their content was not relevant to this review. In other words, despite the fact that the search of PubMed may have identified these papers based on the search terms used, they were in fact not closely enough related to the focus of this work (that is, stakeholder perceptions of generic medicines) to warrant undergoing any appraisal beyond this. The fact that such papers were identified is a function of the design of the searches, using the selected search terms, in a deliberate attempt to ‘capture’ as many published papers in this field as was possible.

The Scopus searches proved less successful, returning 280 articles relevant to patients, 138 articles relevant to physicians and 70 articles relating to pharmacists. These results comprised only publications identified in the PubMed searches, while failing to identify many, and did not add any new publications. Hence, following removal of duplicate findings, there remained 286 articles as stated above.

Additional applicable papers were included if referenced in those found in the above described searches. All of the remaining articles were subjected to critical appraisal and the findings presented below.

### Physicians/general practitioners

Following appropriate exclusion of publications, the literature search returned only 21 publications internationally, within the stated study time period, on the topic of physician perception of generic medicines [15, 16, 31–48], six of which were not in English [34, 35, 39, 41, 44, 46].

Additionally, one further review on the topic of physician opinions of generic medicines was found (referenced in [48]) and included [49]; and one additional article (which was cited in one of the publications found in the systematic review) was further included as it was the only interview-based study that could be found on this topic – a study of GPs’ views of generic medicines by Hassali et al. in Melbourne, Australia [50], based on interviews with ten GPs.

Following determination of applicable publications, those not published in English [34, 35, 39, 41, 44, 46] were excluded. Therefore, a total of 16 papers [15, 16, 31–33, 36–38, 40, 42, 43, 45, 47–50] were reviewed for the physician cohort.

### Pharmacists

Following appropriate exclusion of publications, the literature search returned 11 applicable publications [13–15, 51–58]. No exclusions were made.

### Patients/consumers

Following appropriate exclusion of publications, 36 reports on patient/consumer opinions of generic medicines were published within the defined scope [15, 17–20, 31, 37, 52, 54, 59–85], five of which were not in English [61, 62, 80, 82, 85]. Therefore, 31 papers were reviewed for this cohort.

### Overall

Some publications had scope encompassing more than one cohort (for example, perceptions of physicians and patients), thus an overall total of 52 papers were included in this systematic review; see Table 1 for details, where it can be seen that while 16 papers were found to be relevant to physicians, 11 papers relevant to pharmacists and 31 papers relevant to patients, due to overlap, the number of papers that underwent critical appraisal was 52.

**Table 1:** Summary of studies (sorted by reference number)

Location	Subject(s)	n	Type	Focus	Main findings	Reference
Nigeria	Pharmacists	154	Questionnaire	Perceptions	Many respondents lacked confidence in the quality of the generics available on the Nigerian market, but a majority supported generic substitution practices.	[13]
Czech Republic	Pharmacists	615	Questionnaire	Opinions, attitudes, experiences	A majority of respondents considered generic drugs as bioequivalent and therapeutically equivalent.	[14]
					A small number of pharmacists believed that generic products were of lower quality than branded drugs and expected generics to cause more adverse drug reactions.	
South Africa	Consumers and healthcare professionals	73 consumers	Focus group discussions with consumers	Comparison of healthcare professionals’ and consumers’ opinions, along with testing of generic formulations	All formulations passed *in vitro* tests for quality. Therefore, the study showed clear differences between perceptions of quality and actual quality of medicines, suggesting that implementation of generic medicine policy requires information gaps to be addressed.	[15]
		15 healthcare professionals	Semi-structured interviews with healthcare professionals			
USA	Physicians	506	Questionnaire	Perceptions	A meaningful proportion of physicians expressed negative perceptions about generic medications, representing a potential barrier to generic use. Payers and policymakers trying to encourage generic use may consider educational campaigns targeting older physicians.	[16]
Ireland	Patients	42	Interviews	Perceptions	Variable knowledge about generic medicines among patients. Although patients were supportive of their more widespread use, concerns regarding safety, clinical effectiveness and manufacturing quality of generic medicines were identified.	[17]
Denmark	Patients	2,476	Questionnaire	Attitudes, beliefs, experiences	Patients who had once experienced a generic switch were more likely to accept a future generic switch.	[18]
					Negative views on generic medicines were negatively associated with switching, while beliefs about medicine and confidence in the healthcare system had no influence.	
USA	Female patients	50	Focus groups	Perceptions	Generally favourable perceptions regarding generic drug discount programs.	[19]
					Study participants believed that generic medicines were generally effective and similar to their brand equivalents; however, there was an association between severity of illness and willingness to utilise generic prescription drugs.	
Australia	Patients	47	Postal survey	Attitudes and perceptions to generic substitution with AEDs	Considerable concern was found among patients with epilepsy about generic substitution of antiepileptic drugs.	[20]
					More clinical data and research on bioequivalence of generic antiepileptic medicines may help to address these concerns.	
Canada	Patients and physicians	81 patients	Questionnaire	Interchangeability of warfarin	While most patients and physicians appeared to have accepted the principle of therapeutic equivalence of generic and brand name warfarin, a sizable minority had concerns that could influence prescribing and compliance, believing that generic warfarin was neither as safe nor as effective as brand name warfarin.	[31]
		110 physicians				
Slovenia	GPs	117	Postal survey	Attitudes, generic prescribing	The majority of GPs perceived generics to have the same effectiveness as branded drugs.	[32]
					Slovene GPs were aware of the cost of prescribed drugs. They were willing to accept independent academic detailing to improve their prescribing and were willing to increase prescribing generic drugs under certain conditions.	
Saudi Arabia	Physicians	772	Questionnaire	Perceptions and attitudes, generic prescribing	Most physicians supported generic substitution, but they indicated that there were certain clinical situations where they preferred to use brand name drugs.	[33]
Greece	Physicians	1,204	Postal questionnaire	Perceptions	Physicians seemed to be open to prescribing generic medicines, despite the fact that they did not do so at the time the article was published. The expansion of the generics market should have a positive impact on patients’ access to cheaper drugs.	[36]
USA	Patients and physicians	550 patients, 606 physicians	Online survey	Perceptions in the context of treatment of epilepsy	About half of physicians were extremely/very likely to request that brand AEDs not be substituted with a generic.	[37]
					Perceptions among physicians and patients did not align with the FDA position that generic AEDs have the same clinical effect and safety profile as branded AEDs.	
					More research is needed to determine if generic AEDs are bioequivalent in real-life situations.	
Jamaica	Physicians	60	Questionnaire	Acceptance, perceptions	There were doubts about whether bioequivalence of a generic was equitable to therapeutic equivalence to an innovator drug. A third of the physicians were able to identify at least one case in the past year of clinical problems with generic substitutes, which they perceived would not have occurred with the innovator.	[38]
Pakistan	Physicians	11	Semi-structured interviews	Knowledge, attitudes, perceptions	The major themes identified were knowledge of generic medicines, perceptions regarding generic medicines, attitude towards generic medicines and perception towards marketing strategies of pharmaceutical industry for brands and generics, as well as recommendations to further enhance generic utilisation.	[40]
Pakistan	GPs	206	Questionnaire	Perceptions, attitudes	Close to three-quarters of the respondents showed correct knowledge about generic medicines being a ‘copy of the brand name medicines’ and ‘interchangeable with brand name medicines’. The majority of respondents incorrectly understood that the generic medicines were less safe than brand name medicines. The majority of respondents believed that their prescribing decision was influenced by pharmaceutical company representatives.	[42]
					Knowledge gaps evident.	
Italy	Family paediatricians	303	Online questionnaire	Perceptions, patterns of use	Major issues related to scepticism about reliability of bioequivalence tests and safety of switchability from branded to generic equivalents. More information about generic drugs and more research in the field of paediatric pharmacology are needed to increase the generic medicine prescription rate.	[43]
Malaysia	GPs	87	Postal survey	Knowledge, perceptions	Although it appeared that GPs had largely accepted the use of generic medicines, they still had concerns regarding the reliability and quality of such products. GPs need to be educated and reassured about the generic product approval system in Malaysia concerning bioequivalence, quality and safety.	[45]
Germany	Physicians: psychiatrists	410	Survey	Decision-making between generic and branded	Psychiatrists were more likely to choose branded drugs when imagining choosing the drug for themselves (versus recommending a drug to a patient).	[47]
					Psychiatrists were more likely to choose generic antidepressants than generic antipsychotics.	
Ireland	GPs	34	Semi-structured interviews	Perceptions, beliefs, behaviours	Majority of participating GPs actively prescribed generic medicines. Predominantly, participants believed that generics worked as effectively, and were of the same quality, as originator medicines A minority of GPs were of the view that generics were manufactured to a poorer quality than originators and may be a risk to patient safety.	[48]
USA, Australia, Finland, Malaysia, Slovenia, France, Ireland, UK and Jamaica	Physicians	14 papers	Review article	Views	Physicians lack knowledge of regulatory requirements imposed on generics.	[49]
		1980–2008			Being cheaper than their branded counterparts raised the concerns of the physicians about their quality, safety and effectiveness, especially in the presence of heavy and successful promotional activities from brand name industry.	
Australia	GPs	10	Semi-structured interview	Perceptions	Study suggested that GPs in Melbourne had mixed attitudes to generic prescribing.	[50]
					Also shows that misconceptions about safety and efficacy of generic medicines still persisted among some GPs and that unless they were sufficiently educated by interested parties, such as the government and the generic medicine industry, this will have a negative impact on utilisation of generic medicines in future.	
Australia, Canada, France, Germany and UK	Pharmacists	254	Web questionnaire	Attitudes, interchangeability of dry powder inhalers	Just 6 % of pharmacists considered that dry powder inhalers were interchangeable, with a high level of concern shown about interchangeable use and with patient confusion being the main concern expressed.	[51]
Portugal	Patients and pharmacists	95 pharmacists	Questionnaire	Perceptions, attitudes	More information for patients is necessary. Greater levels of acceptance seen in patients with higher education levels or those who had discussed substitution with physician/pharmacist.	[52]
		417 patients			A majority of patients were willing to accept generics on recommendation of healthcare professional.	
Sweden	Pharmacists	16	Interviews	Experiences, attitudes	Pharmacists found it positive that generic substitution decreased the costs for pharmaceuticals but also emphasised that the switch can confuse and worry patients, which could result in less benefit from treatment.	[53]
					To prevent known confusion and concern among patients, it is important that community pharmacists acquire the necessary tools and knowledge to manage this situation, and communicate effectively with patients.	
USA	Patients, pharmacists	82 patients	Postal survey	Patient and pharmacist knowledge of, and attitudes toward, reporting adverse events due to using generic AEDs	More than 92 % of patients and 85 % of pharmacists agreed that switching between forms of the same AEDs may cause an increase in seizures or adverse effects.	[54]
		112 community pharmacists				
Malaysia	Pharmacists	219	Postal questionnaire	Views	Only 50.2 % of the surveyed pharmacists agreed that all products that were approved as generic equivalents can be considered therapeutically equivalent with the innovator medicines. The Malaysian pharmacists had a lack of information and/or trust in generic manufacturing and/or approval system in Malaysia.	[55]
New Zealand	Pharmacists	360	Postal questionnaire	Views, knowledge	70 % of pharmacists stated there was no difference in safety between original brand and generic medicines.	[56]
					65 % stated that original brand medicines were of higher quality than their generic counterparts, and half stated that generic medicines and original brand medicines were equally effective.	
					Concerns were raised regarding quality, safety and effectiveness; however, most of the pharmacists acknowledged the economic benefits to the healthcare system.	
France	Pharmacists	1,000	Postal survey	Opinions, behaviours	90 % of the pharmacists were favourable to the implementation of generic substitution. 42.5 % declared they systematically offered patients the generic drug, whereas 55 % chose to target specific populations for substitution.	[57]
Ireland	Community pharmacists	44	Semi-structured interviews	Perceptions, attitudes	Only a small number demonstrated some reticence regarding generics.	[58]
					89 % of pharmacists reported receiving patient complaints regarding use of generic medicine, although 64 % suggested that this was due to a nocebo effect (that is, a result of patients’ preconceived notions that generics were inferior). Only a minority (21 %) reported that they had attempted to educate patients as to the equivalency of generics.	
Germany	Patients	804	Survey	Perceptions	GPs were in an ideal position to adequately inform their patients about the equivalence of brand name and generic drugs. Patients held views that inexpensive drugs must be inferior.	[59]
Norway	Patients	281	Written questionnaire	Experiences, attitudes	36 % of the patients reported negative experiences after medication substitution.	[60]
					Generic drug substitution was not considered an equal alternative to branded drugs by a number of patients for whom additional information and support may be needed.	
Norway	Hypertensive patients	174	Interviews	Challenges of generic substitution in adherence	One in three said generic substitution made keeping track of their medications more demanding.	[63]
					A negative attitude towards generics was significantly associated with low educational attainment, an increasing number of drugs, having general concerns about medicine use and having received insufficient information regarding generic substitution.	
Finland	Patients	256	Questionnaire	Preferences	Approximately half of the respondents were strongly price-sensitive, while the others had other preferences such as brand or an opportunity to buy the medicine at a pharmacy, or to have a physician or a pharmacist as an information source.	[64]
USA	Patients	356	Postal survey	Perception of generic AEDs	A significant percentage of patients reported that generic AEDs were responsible for breakthrough seizures and increased side effects. A significant percentage of patients also reported switching back to a brand name AED and expressed concern over pharmacies switching to generic AEDs without a patient’s or physician’s consent.	[65]
The Netherlands	Patients	106	Interview	Attitudes to substitution, oral atypical antipsychotics	3 % stated that they would be unlikely to take a generic antipsychotic if their pharmacist were to substitute it.	[66]
					Patients with psychoses/schizophrenia perceived generic versions of their antipsychotics as being significantly different. This perceived difference lowered their intention of continuing to take the medication, thus possibly jeopardising treatment outcome.	
USA	Patients	971	Postal survey	Relationship between beliefs and generic usage	Generic drug use was most closely associated with communication by providers about generics resulting in comfort with generic substitution.	[67]
USA	Patients	1,054	Postal survey	Perceptions	Patients agreed that generics were less expensive and better value than brand name drugs, and were just as safe.	[68]
					Findings may indicate that perceptions about generic essential medications have improved over time. Efforts to educate patients could positively influence usage of generics.	
New Zealand	Consumers	441	Questionnaire	Knowledge, perceptions, attitudes	Pharmacists were the main source of information regarding generic medicines followed by doctors and media.	[69]
					A higher level of education had a direct relationship with having correct knowledge of generics.	
					Many consumers have misconceptions regarding generic medicines.	
Australia	Patients: senior citizens	104	Focus groups	Perceptions	Demonstrated considerable mistrust of generic medicines. Participants highlighted their uncertainty about the extent of pharmaceutical companies’ influence on health professionals, the mistrust of foreign generic manufacturers and scepticism in their equivalence.	[70]
South Africa	Consumers	73	Focus groups	Perceptions	Irrespective of socioeconomic status, respondents described medicine quality in terms of the effect the medicine produced on felt symptoms.	[71]
					Generic medicines were considered to be poor quality and treated with suspicion.	
					Cost, avoidance of feeling ‘second class’, receiving individualised care and choice in drug selection were the main determinants influencing procurement behaviour.	
Norway	Patients: Pakistani immigrants	83	Interviews	Challenges following generic substitution	One-quarter of the participants were of the opinion that cheaper generic drugs were counterfeit drugs. Two-thirds had accepted generic substitution in the pharmacy, whereas the remaining participants had either opposed or were unaware of the substitution.	[72]
Finland	Public: consumers	1,844	Postal survey	Opinions	Finnish consumers considered generic substitution a good reform. They also had confidence in the effect of cheaper medicines. Savings were the main reason for accepting generic substitution.	[73]
					Substitution was not considered to cause any risk to drug safety.	
					Two main reasons for substituting were a desire to save money and recommendation by pharmacists.	
					Female gender, older age and use of prescription drugs were associated with refusing.	
Japan	Patients	1,215	Questionnaire	Attitudes to generic substitution	The public awareness program on generic drugs should be expanded to include more detailed information so that patients obtain the correct understanding of generic substitution. It is critical that physicians and pharmacists have the proper understanding of generic drug substitution and provide the correct information to patients.	[74]
Iraq	Consumers	14	Face-to-face interviews	Perceptions	Most of the participants understood that generics cost less compared with their branded counterparts, and their physicians and pharmacists had given them information on generics.	[75]
					However, knowledge of generic medicines was lacking among consumers in Iraq and their primary reason for using generic medicines was that they were less expensive.	
Finland	Public	1,844	Postal questionnaire	Opinions on refusal of generic substitution	Main reasons for generic substitution refusal were satisfaction with their current medicine and/or that a decision on a drug product had been made in co-operation with their physician. Most of these individuals indicated that they would be unwilling to accept generic substitution in the future.	[76]
USA	Patients	172 female patients of childbearing age	Oral questionnaire	Beliefs, perceptions	Awareness of the benefits of generics did not equal preferences for personal use of generics.	[77]
					About a quarter believed that brand name medications were more effective than generics.	
					13.4 % believed that generics caused more side effects.	
USA	Patients	30	Focus groups	Perceptions, barriers to use	Barriers to generic medication use included: perceptions that generics are less potent than brand name medications, require higher doses and, therefore, result in more side effects; generics are not ‘real’ medicines; generics are for minor but not serious illnesses; the medical system cannot be trusted; and poor people are forced to ‘settle’ for generics.	[78]
UAE	Renal patients	188	Survey	Views on generic substitution	70 % of patients were aware of the availability of generic medicines.	[79]
					31 % felt that generics were not equivalent or only sometimes equivalent to branded medicines. Nearly half the patients stated they would refuse generic substitution when it became available if this was just to save the health authority money.	
Germany	Patients	126	Questionnaire	Perspectives to substitution in context of treatment of epilepsy	32 % of the patients who already experienced a switch to generic AEDs complained of problems with the switch. However, patients who had never switched were more concerned about generic substitution than those who had already switched.	[81]
USA	Consumers	183	Survey	Factors influencing purchasing	Single most influential factor was lower cost.	[83]
					Other factors, including advertisements, duration of the OTC drug effectiveness, severity of sickness, preferable form of OTC medication, safety of the OTC, relief of multiple symptoms and preferred company, would persuade consumers to pay more for brand name drugs.	
USA, Europe, Canada, Australia, Brazil and Malaysia	Consumers	20 studies	Review article	Views, chronological	Mixed reactions, related to development level of country.	[84]
					However, reasonably positive attitude (40–60 %) stable across studies.	
		1970–October 2008			Positive attitudes not necessarily translated into increased use of generic products.	

*AED* antiepileptic drug, *FDA* US Food and Drug Administration, *GP* general practitioner, *OTC* over-the-counter

### Review of reported methodologies

The methodological approach most commonly observed in the research appraised in this review was the self-administered questionnaire or survey, where dissemination either by post or online methods appeared to be the most frequent routes of questionnaire provision to participants. The conducting of such research by qualitative means (for example, by interview or focus group) was also identified, albeit that such reports were found in smaller numbers.

It is notable that only three physician-specific qualitative research papers were found: studies of GPs’ views in Pakistan [40]; Australia [50]; and Ireland [48], which described the outcome of interviews with 11, 10 and 34 participants, respectively.

In the case of pharmacists, only two interview-based studies were found: 16 participants were interviewed for a study in Sweden [53]; and 44 for a study in Ireland [58]. In research into patient views, five interview-based studies of the opinions of patients were found from Iraq [75], The Netherlands [66], Norway [63, 72] and Ireland [17], which interviewed 14, 106, 83, 174 and 42 participants, respectively. Four focus group studies from South Africa [71], the USA [19, 78] and Australia [70], which had 73, 30, 50 and 104 participants, respectively, were found. Additionally, one mixed-subject qualitative study, which conducted focus groups with 73 consumers and semi-structured interviews with 15 healthcare professionals (six each of which were physicians and pharmacists), compared consumers’ and professionals’ opinions, along with *in vitro* testing of a small number of generic formulations in South Africa [15].

In summary, the greatest numbers of studies found were quantitative assessments focused on the patient/consumer cohort. As is often found in quantitative studies, the number of participants was comparatively higher than in the qualitative studies of patient and both professional groups. However, when viewed collectively, the complementary methodologies employed by the various research groups have provided a reasonable breadth and depth of insight into the knowledge of, and perceptions, opinions and behaviours towards, generic medicines within the patient, physician and pharmacist subject cohorts.

### Review of stakeholder opinions

#### Physicians

One of the articles found was a comprehensive and clearly presented narrative review, by Hassali et al. [49], which collated international studies published between 1980 and 2008. This article coalesced the collective views of physicians as accepting of generic substitution (GS) under policy and economic pressures, but having concerns regarding the overall quality, reliability and switchability of generic drugs. This review further theorised that those concerns may prevent full adoption of generic drug prescribing and substitution by physicians, which could lead to escalation in healthcare costs for governments, insurers or consumers directly [49].

This critical appraisal has gone further and has developed second-order constructs from the 17 included articles. During the appraisal process, it became clear that the articles could be defined as belonging to seven specific, non-mutually exclusive groups related to: a) physician reservations regarding generic medicines [15, 16, 43, 45, 47, 48, 50]; b) physicians’ confidence in their level of knowledge and understanding of generic medicines and associated topics [15, 31–33, 38, 40, 48, 50]; c) reference by physicians to use of pharmaceutical industry source of information regarding generic medicines [16, 32, 33, 36, 42, 45, 50]; d) physicians’ perceived influence of the pharmaceutical industry and company representatives [15, 16, 32, 33, 36, 37, 40, 42, 45, 47, 48, 50]; e) physicians’ experience of financial incentives provided to physicians to influence prescribing behaviour [15, 36, 40, 45, 47, 48, 50]; f) physicians’ experience of pressure applied by patients regarding branded products [15, 16, 32, 33, 36, 40, 48]; and g) physician belief that education (specifically regarding aspects of bioequivalence) is required for greater use of generics in their market (all papers). This classification emerged only as part of this appraisal; it was not commonly used before then by authors of any of the individual articles or in the aforementioned review by Hassali et al. [49].

More specifically, with respect to a) above, in the studies describing reservations expressed by physicians (and other healthcare professionals), specific references were made to: lack of confidence in foreign manufacturers, particularly those in India and China [45, 48]; doubts about equivalence [16, 33, 38, 42, 47, 48, 50]; and the expression of personal preference for branded medications if required for themselves [16, 47, 48].

Other, less common attitudes expressed related to physicians reporting breakthrough seizures associated with GS of an antiepileptic drug (AED) and as a result many were likely to prefer that generic AEDs were not used [20, 37, 48]. Significant proportions of physicians express a preference for brand name medications both generally [31, 48] and in the case of some specific medications (in this instance, warfarin) [38]. Also, older physicians were more likely to have a poorer opinion of generics [16, 48]; however, despite these stated misgivings, a majority of physicians were largely accepting of the use of generic medicines [32, 33, 36, 42, 45, 48].

While some physicians have expressed an expectation that generics should be cheaper than they are [32], many state the lower cost of generics (and a consideration of the patient’s ability to pay) as one of the main factors affecting their prescription of these medicines [15, 38, 45, 48]. Other factors affecting use include knowledge and reliability of the manufacturer [15, 45, 48] and the illness being treated [47, 48].

All of the studies appraised were included based on a subjective assessment of their applicability to the topic, using the criteria outlined earlier. It was further thought that, given the low number of studies available, an inclusive approach would lead to a greater chance of capturing all potentially relevant perspectives.

Given the paucity of literature on the topic of physician attitudes towards generic medicines, it is unsurprising that each of the papers contributes some new information, albeit specific to particular countries or regions (Table 1) and, as such, the published outcomes may not be readily generalisable. It is also notable that with relatively few exceptions [15, 36, 40, 42, 50] the researchers utilised questionnaires comprised of closed questions, many of which relied on Likert scale-type responses. In almost all cases, the study instruments were assessed for face validity and pilot tested. However, only one of the papers reported specific efforts to ensure appropriate reading level [31], while in three studies [15, 16, 37] payment was provided to physicians participating. Indeed, in one of the studies [37], the payment considerably altered the expected response rate, acknowledged by the authors who however stated that they believed the payment did not affect the nature of the responses provided. It was also noted that with the exception of a single study [31], no data were presented as to whether the participating physicians freely communicated their perspectives on generic medicines to their patients, a factor that may influence subsequent consumer attitudes and behaviour.

Across the 17 papers appraised, varying recruitment strategies were employed, with a number of the research groups stratifying participants, albeit with a wide range of response rates. However, recruitment biases were evident in a subset of these [31, 37, 38, 40, 42, 45], with the most challenging bias involving recruitment of participants based on databases of suitable practitioners provided by representatives of the pharmaceutical industry [42].

On a positive note, one study assessing the attitudes and underlying rationales of psychiatrists towards generic products [47] deserves mention. The authors, recognising a potential bias risk associated with the fact that all participants were attendees at a single conference, established elaborate vignette-based scenarios, with multiple potential questioning delivered to a large number of participants, enabled further by sophisticated statistical analyses.

#### Pharmacists

Assessments of pharmacist perceptions of generic medicines have been carried out in a relatively limited number of countries since 2003: New Zealand [56]; Portugal [52]; South Africa [15]; Malaysia [55]; France [57]; Ireland [58]; and Sweden [53], and also in relation to specific medications such as antiepileptic formulations [54] and inhalers [51]. In fact, as the 11 studies found here appear to be the only published investigations on the topic of pharmacist perception of generic medicines, this appears to be a relatively underexplored area, internationally.

While critically appraising the papers found, and attempting a synthesis of their findings, it was evident that, compared to the physician-focused reports critiqued above, there was considerably less consensus regarding potential second-order constructs. To interpret, present and discuss the findings of the papers, the chosen approach was to identify synthetic unifying themes (an approach described by Fleming [86]). The four unifying themes identified, comprised of sub-themes or issues, are detailed in Table 2. The themes are:

- Pharmacists’ concerns regarding patient understanding of generic medicines and substitution, patient safety and compliance with treatments (8/11 papers).
- Pharmacists’ understanding of generic medicines and substitution, and pharmacists’ confidence in quality, efficacy and safety of generic medicines (7/11 papers).
- Practical aspects of pharmacists’ practice as affected by generic medicines and substitution (10/11 papers).
- Pharmacists’ suggestions to improve generic medicine use and education of stakeholders regarding this (10/11 papers).

**Table 2:** Unifying themes and contributing sub-themes from pharmacist papers

Theme	Sub-themes	Examples of contributing papers
Pharmacists’ concerns regarding patient understanding of generic medicines and substitution, patient safety and compliance with treatments	a. Patient confusion	[14, 51–53, 56, 58]
	b. Concerns regarding interchangeability	[13, 14, 51, 53, 54, 56, 58]
	c. Problems with patient compliance	[13, 51, 53, 56]
Pharmacists’ understanding of generic medicines and substitution, and pharmacists’ confidence in quality, efficacy and safety of generic medicines	a. Level of confidence in knowledge	[15, 53, 55, 56, 58]
	b. Hospital-based pharmacists versus community pharmacists	[13]
	c. Need to contact prescriber	[51]
	d. Belief that the patient prefers physician opinion	[52, 53, 58]
	e. Requirement for adverse event reporting	[54, 56]
Practical aspects of pharmacists’ practice as affected by generic medicines and substitution	a. Financial incentives by pharmaceutical industry	[13, 15, 52, 56–58]
	b. Increased pharmacist workload	[13, 51, 53, 54, 56, 58]
	c. Adverse effect on stocking levels	[14, 51, 53]
	d. Influence of industry representatives	[15, 52, 58]
Pharmacists’ suggestions to improve generic medicine use and education of stakeholders	a. General education needed	[14, 15, 52–58]
	b. Patients should have a role in medication decision	[51, 53]
	c. Need for change in prescribing patterns	[52, 53, 57, 58]

Interpreting these perspectives, it appears that pharmacists tend to hold mainly positive views of generics with respect to their safety and equivalence and support for GS (for example, German pharmacists were overwhelmingly cautious (albeit that only dry powder inhalers were discussed in this study; it is also worth noting that this study was supported by GlaxoSmithKline who may have a vested interest in the outcomes of such research) [51]; and while the French and Irish counterparts were mainly positive [57, 58]), for some pharmacists there remains cause for concern [15, 51, 55, 56]. Of these, one of the primary concerns relates to patient safety and, explicitly, the potential for confusion to be caused, particularly in the case of older patients, due to differing appearance and presentation of generic medicines, which has been reported to have an impact on medication compliance [51, 53]. In fact, two suggestions made by pharmacists to potentially mitigate risk in this area were: a) that patients be informed and involved in the decision-making process regarding the selection of medicinal products provided to them [51]; and b) that an upper age limit be established such that older patients (possibly more prone to confusion) would not encounter unfamiliar medications [53]. Studies have reported perceptions of increased pharmacist workload associated with generic medicines, including provision of generic medicine-related information to patients and variations in stock management [58]. Possibly related to these pragmatic topics, one of the primary pharmacist recommendations was for increased stakeholder education regarding generic medicines and substitution. Further, in one study addressing both patient and pharmacist perspectives, 78 % of patients believed themselves to be well-informed regarding generic medicines while 83 % of their pharmacists perceived a lack of patient understanding [52]. However, notably, recommendations regarding education are not all focused on patients and include measures to be taken to ensure that the pharmacist group have the correct knowledge in order to aid and facilitate this knowledge transfer to patients [15, 53, 55–58, 74]. That said, some pharmacists believe that advice given to patients, by pharmacists, regarding their medications is less valued or trusted than advice from prescribing physicians [52, 53].

Each of these 11 papers represented efforts made to elucidate the views and behaviours of pharmacists. As shown in Table 2, most of the research groups involved made efforts to design their study instruments based on key opinion leader advice and, in the majority of cases, the instruments were piloted before use.

However, 8 of the 11 papers failed to protect against recruitment bias, with one dominated by pharmacy owners who may be influenced by profit margins achievable through dispensing originator medicines and attractive industry bonusing/reimbursement [55]. Another (originating from South Africa) [16] described an assessment in which both perceptions and experience of generic medicine quality in South Africa were compared with actual generic medicine quality. Given the relatively poor reputation of generic medicine manufacture in South Africa (as stated in the paper itself), the outcomes were predictable and did not add any new information to the field in that context. The transferability of the study outcomes beyond South Africa is further hampered by the authors’ acknowledgement that due to their purposeful sampling in urban (affluent) areas of South Africa, their results may be confounded by additional negative biases attributable to those particular communities and population demographic. While other papers also focus on specific population groups or countries, it is the combination of attitudinal perspectives with evaluation of experience of actual specific defined products that limits transferability in this case.

A common theme in pharmacist-oriented papers was a focus on the negative connotations of generic medicines as experienced by pharmacists. Only one study [52] reported incidence (albeit low) of patients requesting generic medicines rather than the more frequently reported reticence of patients. It would be interesting to meter such incidence as recorded or perceived by pharmacists and to compare those data with patient-reported measures. A final comment relates to a possible ‘missed opportunity’ throughout these studies, whereby study instruments have explored pharmacists’ perceptions and experiences of generic medicine efficacy, safety and quality, but only 2 of the 11 studies followed this line of thought by determining what actions the pharmacists then took with respect to adverse event reporting, an important facet of pharmaceutical regulation that relies on post-market monitoring of products (originator or generic) to ensure that patient safety is assured.

#### Patients

Patient-focused studies have, relative to the opinions of healthcare professionals, had comparatively more attention, internationally. In summary, published reports have originated in Norway (patients attitudes to generic substitution) [60], Finland (preferences of patients for generic and branded over-the-counter (OTC) pain medicines) [64], Portugal (patient perceptions of underuse of generics and their attitudes towards generic substitution) [52], South Africa (consumer perceptions of generic drug quality compared with actual drug quality) [15, 71], New Zealand (patients’ perceptions, knowledge and attitudes regarding generic medicines and investigation of patients’ attitudes towards generic substitution of oral antipsychotics) [66, 69], Iraq (consumers’ knowledge relating to generic medicines) [75], the USA (patient knowledge of, and attitudes relating to, formulation switching of antiepileptic drugs) [54] and Ireland (patient perceptions of generic medicines) [17], amongst others.

In critically appraising the papers found, and attempting a synthesis of their findings, it was evident that, compared to the pharmacist-focused reports critiqued above, there was greater consensus regarding potential second-order constructs. To interpret, present and discuss the findings of the papers, the chosen approach was to identify synthetic unifying themes [86]. The unifying themes identified, comprised of sub-themes, are detailed in Table 3. The themes are:

- Patients’ lack of confidence in generic medicines, contributed to by initial scepticism, provision of poor or poorly understood information, and concerns regarding packaging and/or appearance of generic medicines (18/30 papers).
- Patients’ actual experiences in using generic medicines, not exclusively negative, including difficulties associated with treatment adherence or compliance (9/30 papers).
- Factors influencing patient acceptance of generic medicines, including patient involvement in decision-making, age, income and severity of illness (7/30 papers).
- Provision of information and education regarding generic medicines (10/30 papers).

**Table 3:** Unifying themes and contributing sub-themes from patient/consumer papers

Theme	Sub-themes	Examples of contributing papers
Patients’ lack of confidence in generic medicines	a. Scepticism	[18, 20, 59, 63, 65, 66, 68–72, 76–79, 81]
	b. Provision of poor or poorly understood information	[19, 59, 63, 70, 77–79]
	c. Concerns regarding packaging and/or appearance of generic medicines	[59, 63, 65, 66, 69, 70, 75]
Patients’ actual experiences in using generic medicines	a. Poor experience	[59, 60, 63, 65, 72, 76, 81]
	b. Not exclusively negative	[18, 59, 60, 65, 66, 73]
	c. Difficulties associated with treatment adherence or compliance	[63, 66, 70, 72, 75, 81]
Factors influencing patient acceptance of generic medicines	a. Patient involvement in decision-making	[19, 20, 65, 66, 76]
	b. Advanced age incompatible with generic use	[60, 73]
	c. Income/education level	[68, 69, 77]
	d. Severity of illness/drug type	[19, 63, 68, 69, 77, 78, 81, 83]
	e. Nocebo	[17, 59]
Provision of information and education regarding generic medicines	a. Education and support	[19, 60, 63, 66, 68, 72, 74, 75, 79, 83]
	b. Source of information, including physician versus pharmacist	[70, 71, 78]

In a 2009 review article on patient views of generic medicines (reviewing literature up to October 2008), Hassali et al. [84] determined that patient confidence and knowledge had improved steadily since the 1970s, with the greatest levels of acceptance being seen in developed countries. This growth in confidence was ascribed to mass educational efforts and greater communication amongst healthcare professionals and patients, although safety and efficacy were stated as being the main barriers to acceptance of generic substitutions. Hassali et al. stated that consumers with lower educational levels tend to have greater mistrust in generics, and this appears to continue to be the case, as reported in more recent research [52, 63, 69]. Interestingly, however, this did not appear to be the case in a study published by Kohli and Buller in 2013, who stated that even though their study population had lower socioeconomic status and education, more than half of respondents reported choosing generic drugs rather than brand name drugs [83]. When delved into more deeply, the apparent disparity is somewhat explained by the fact that while the latter study is broadly similar to the other three detailed above with regard to methodology and number of participants, the focus is solely on generic OTC medicines. In fact, the authors found that, similar to the Finnish study above (which was inclusive of OTC medicines), lower cost and number of doses in the package were important factors that respondents rated as having substantial influence on their purchase of generic OTCs. In addition, factors that were determined to have no statistical significance in influencing consumer purchasing patterns included advice from healthcare provider, advice from family and friends, look of the package, degree of sickness (mild), taste of the OTC and greater effectiveness of the OTC. It is noteworthy though that the authors did not survey participants about their health insurance status and whether they received coverage for OTC medications.

The authors of the 2009 review recommended that further research in the area of patient views should be focussed on developing countries, where cost savings are more urgently needed; and in fact research published since 2009 has been more focussed on developed countries, possibly as a result of where research funding is available to investigate consumer opinions.

The greatest level of poor opinion and mistrust in generics is seen in the patient rather than healthcare professional cohort. They report negative perceptions in general as well as in specific terms. In general terms, views are reported such as: being largely unwilling to accept, or having mistrust in generics [70, 76]; having had negative experiences following a generic substitution [60, 72]; mistrust of foreign manufacturers [70]; and a belief that cheaper equals inferior [17, 59]. In specific terms, patients reported breakthrough seizures following substitution of an AED [20, 37, 65, 81], reported being advised by a physician not to accept GS for an AED R61 and are of the opinion that poor people are forced to ‘settle’ for generics [59, 78] (in one study nearly half of the respondents stated that they would refuse a GS if it was only to save the health system money [79]). This reported behaviour is supported by beliefs that generic medicines are believed to be poor quality, are treated with suspicion and are considered ‘second class’ within this cohort [17, 66, 71].

Many patients do not consider generic medicines equivalent to the branded product [60, 63, 70, 72, 78, 79] and there is also a belief that brand name medications are more effective/potent and have fewer side effects [63, 72, 77, 78]. Furthermore, patients appear to be more accepting of generics for treatment of minor illnesses but prefer branded medicines for more serious health problems [19, 69, 78, 81]. Links between GS and lack of compliance with taking medication can also be observed: patients have reported that GS made it more demanding to keep track of their medication [63, 72] and that variability in packaging or appearance caused issues [17, 70]. Worryingly, in one study patients were seen to be taking two or more equivalent medications, concurrently, due to lack of understanding, following a GS [72]. While many misconceptions are held within the patient group [69], one particularly disturbing association can be observed in the literature: some consumers held the belief that generic and falsified (that is, counterfeit) medicines are the same [15, 72].

There are, however, several studies that report positive views in the patient group. In a Finnish study a majority of patients indicated that they did not notice any difference following a GS [73]. Other studies have shown patient groups that do not consider generics to pose a safety risk [17, 68, 73], that they generally accept generics as being equivalent [19] and in one study only a minority believed that brand name medicines were better than generics [68]. While perceptions appear to be improving over time [68], recent literature in the area of patient perceptions has a common recommendation running through it: the continued need for effective information to be communicated to consumers and for trusted healthcare professionals to take time to provide clear education about the equivalence of generic formulations [15, 17, 52, 60, 63, 68, 77, 78]. Knowledge gaps, often considerable, exist within this cohort [17, 74, 75, 78]; hence education is seen as a key factor to improvement of confidence in, and therefore usage of, generic medication. In support of this, one study found that the provision of a short explanation was seen to have a positive effect on patient likelihood to accept a generic [66]. Whether accepting or not, patients wish to be informed as to their healthcare matters, with a view amongst patients that a GS should not take place without their being informed [17, 65].

While the lower cost of generics is the primary incentive associated with their use [52, 60, 64, 73, 75, 83], it is interesting that an awareness of the benefits of generics does not always translate into a preference for their use [76, 77]. Indeed, a study in Finland [73] showed that while a majority of patients stated that more generics should be used, they did not exhibit a preference to use them themselves.

There appears to be considerable evidence that patients who have had a previous good experience with a generic medication are more likely to accept generics in the future [15, 18, 52, 67, 69, 74] and, as patients who have never experienced a GS appeared to be more concerned about taking a generic than those who had [81], this reinforces the importance of the role of healthcare professionals and of provision of accessible information to the patient cohort. In fact, several studies have reported patient trust in healthcare professionals and their acceptance of recommendations of generic medicines, by trusted professionals, despite their own lack of confidence in GS [17, 52, 66, 71, 73–75].

In critically appraising these 30 studies, particular attention was paid to the quality of the study design, the analysis and interpretation of the data, as described by the authors, and to the conclusions drawn. A number of potential confounding factors were evident:

- In the majority of the studies, there was an apparent bias towards investigating negative connotations of patient perceptions of generic medicines. Only a subset of the studies investigated the positive experiences of patients, and whether they had preferences for or had habitually requested generic medicines over originator products [19, 20, 66, 68, 74, 77, 81, 83]. In one specific case, data are presented only for patients who had declined recommended generic medicines [76].
- In determining attitudes and perceptions of patients, many of the studies relied on self-reported questionnaires. In doing so, there is an assumption made that the information provided by the patients regarding their exposure to generic products and, indeed, their stated illnesses are factual. Only four of the research groups correlated patient-derived information with patient records [18, 67, 78, 79].
- In almost all cases, the authors failed to comment on the potential impact of incomplete/non-returned questionnaires and the information that they may have provided. However, in one specific case, an assumption is made that non-return of data equated to dissatisfaction with generic medicines, leading to a statement that a third of patients held that view [59]. This is problematic, as the missing data corresponded to almost 50 % of the ‘dissatisfied’ cohort. The researchers would have been more accurate in stating that ‘… approximately one-sixth of patients explicitly stated dissatisfaction’.
- Many of the studies use convenience samples raising a query regarding the generalisability of the results to the specific population being evaluated; indeed, transferability of much of the data are hampered by a lack of information regarding participant socioeconomic status and educational background. Similarly, many of the studies focus on narrowly defined cohorts such as those on specific medications (for example, antihypertensive, antiepileptic) or culturally discrete communities (for example, immigrant Pakistanis in Norway, post-apartheid older patients in South Africa or poorly educated black females in rural areas of southern states of the USA).
- Validity is questionable in reports with low response rates (for example, 6 % [20]).
- Payment for participation is recorded in two papers, with some indication that the responses obtained from participants may have been different from those expected, possibly due to wishing to provide perceived acceptable answers [19, 78].
- Especially pertinent to the context of this review, almost all of the studies failed to take into consideration survey readability (for example, Flesch Reading Ease or Flesch–Kincaid Grade Level assessments) and the influence of that on participant responses.

## Discussion

This review is the first (to the authors’ knowledge) to include the views of all three of the main stakeholders in generic medicine usage (prescribers, dispensers and consumers). Quantitative studies have been the main approach taken in determining the views and behaviours of the cohorts in the past. These are generally in the form of self-administered questionnaires/surveys, either online or by post. Qualitative studies [15, 17, 19, 53, 72, 75, 78] were also identified, albeit that such reports were found in smaller numbers (only 7 of the 50 publications made use of qualitative methods). These studies have made a significant contribution, however, in showing, for example, that providing patients with a short explanation about generic medicines had a significantly positive effect on their willingness to take them [66], reinforcing the perception that there remains a need for information provision and education. For example, one qualitative study referred to in this review has shown that correct understanding of generic medicines by the general public, as determined by previous quantitative, survey-based studies, may be overestimated (that is, confusion in the patient cohort between the words ‘generic’ and ‘genetic’) [17]. However, irrespective of choice of qualitative or quantitative approach, similar trends in opinions and beliefs held have been reported.

In all three cohorts, it appears that opinions of generic medicines have improved over the years but that some mistrust appears to remain, most particularly in the patient group. Our observations in this regard complement the findings of a recently published review of the knowledge and perceptions of patients regarding generic medicines [87]. Both studies show that patients tend to prefer branded medications, that they have insufficient knowledge and information about generics (thus leading to the need for appropriate educational interventions), and that physicians and pharmacists play a key role in the promotion of generic medicines to patients and in patients’ acceptance of generic substitution.

The physician group shows some level of lack of confidence, although not to the same extent as consumers. It is interesting to note that many comments made by physicians with regard to lack of confidence in generic medicines (as reported earlier) appear to be in contradiction with the literature, where many examples of the use of generic medicines with no negative clinical impact are provided. In fact, a systematic review which summarised the clinical evidence comparing generic and brand name drugs used in cardiovascular disease, which included randomised controlled trials (RCTs) on narrow therapeutic index drugs such as warfarin, concluded that while the evidence does not support the notion that brand name drugs used in cardiovascular disease are superior to generic drugs, a substantial number of journal editorials counsel against the interchangeability of generic drugs [24]. This contradiction in the literature may go some way to explaining physician confusion and lack of trust in generics. However, it is important to remain cognisant that while there is much evidence to support equivalence and interchangeability in many areas of medicine, there are areas where substitution should continue to be approached with caution, for example, in the prescribing and usage of AEDs.

Pharmacists exhibit the greatest degree of positive opinion, and acceptance, of generic medicines. Such mistrust is likely to be substantiated, and possibly furthered, by recent investigations by the FDA which showed that medications previously designated as equivalent and interchangeable were in fact not therapeutically equivalent; for example, bupropion (trade name Wellbutrin) [88] and more recent investigations into the therapeutic equivalence of methylphenidate hydrochloride generics (trade name Concerta) [89]. It is worth noting that these issues were both seen in extended release preparations (with no issues reported for immediate release formulations) and that concerns with modified or extended release preparations have been described elsewhere [90, 91].

There tends to be agreement that provision of education – particularly, but not exclusively, to the patient cohort – is one of the key factors to improving confidence in, and hence usage of, generic medications. A common finding is that acceptance of generics appears to be higher in consumers with higher levels of education [52, 69] and patients from lower socioeconomic demographics, hence having lower levels of education, tend to have greater mistrust of generics [84], with the exception of the findings of Kohli & Buller in 2013 [83], discussed earlier. However, there may be some bias amongst researchers in focusing on negative connotations while disregarding the equally interesting area of patient acceptance and even, possibly, preference for generic medicines. Indeed, when investigated, positive attitudes were seen to arise due to good experiences with generic medicine use or for economic reasons.

There appears to be a strongly held belief, particularly in the patient group, that less expensive equals lower quality [17, 60, 77], reinforcing the need for ‘myth-busting’ education. While consumers may hold that opinion due to experience with other consumer products, the same principles do not apply to pharmaceuticals because of the highly regulated nature of medicine manufacture and marketing approval requirements. Provision of education on these topics to both consumers and physicians (who have exhibited a lack of knowledge in the regulation of medicines in some publications [32, 45, 49]) could be instrumental to improving confidence in generics by providing greater understanding as to why, in the case of generic medicines, lower prices do not equate with poorer levels of quality or efficacy. However, it is interesting that while patients value the opinions of, and information provided to them by, physicians and pharmacists, there is some patient preference for physician-sourced guidance (for example, in South Africa [71]), which is a factor also commented on by some pharmacists, as discussed earlier.

Of potential importance to policy makers is the on-going trend, reported in the 2009 review [84] and again in a 2013 study [83], that positive attitudes towards generics amongst the consumer group do not necessarily translate into increased usage of generic products.

While acknowledging the fact that very few studies will be perfect in both design and completion, and that contingencies in implementation of study protocols can mar any project, the critical appraisal of the studies included here highlights some points that may confound or limit the data they generated. In summary, however, some of the physician-oriented papers involved recruitment biases, the most notable of which were due to use of pharmaceutical industry-derived databases for participant selection [14] and payment to physicians for their inclusion in studies [9, 16, 18]. With respect to pharmacist-oriented papers, there were again recruitment biases in some reports, mainly due to relatively limited geographic distribution of participants, and a potentially dominant focus on reporting negative connotations of generic medicines while, arguably, missing opportunities to report and discuss positive attributes of generic medicines and substitution from the pharmacist’s perspective. Given that this appraisal involved comparatively greater numbers of patient-focused studies than those featuring perceptions of other stakeholders, it is perhaps reasonable that the greatest number of confounders were determined in the larger collection. Specifically, in the majority of the studies, there was an apparent bias towards investigating negative connotations of patient perceptions of generic medicines. Validity, transferability, participant selection and understandability of the study instruments used have also been discussed in earlier sections, and may in some way challenge the outcomes of the studies. However, it must be acknowledged that in most of the studies appraised, the authors attempted to understand these factors and how they may have influenced their data generation, analysis and interpretation of outcomes.

### Recommendations

Further research may be needed in the area of pharmacist opinions as they have a direct impact on patient acceptance of generic medication and, relative to the other two cohorts, very little attention has been paid to this group.

Contradictions and inconsistencies in the literature need to be addressed, such as the apparent inconsistency between evidence of equivalency reported in many clinical trials and journal editorial counselling against the interchangeability of generics in the treatment of cardiovascular disease, as reported by Kesselheim et al. in their 2008 systematic review [24]. Addressing this would help to improve understanding and provide clarity to healthcare professionals (whose opinions may be negatively affected by conflicting and confusing information), and may, in turn, help to increase trust and confidence in generic medicines within this cohort.

A considerable amount of research in the area of equivalence of generic medicines has shown that generics can be used safely with no negative clinical impact [23–27, 92]. However, some discrepancies remain between patient and professional experiences and the stated equivalence by regulators [43]. While acknowledging that there are difficulties in some areas (for example, with AEDs [20, 37] or the differences in excipients or salts used in generic formulations [90]), education of healthcare professionals on the content of the many studies, which demonstrate that there are no clinical differences between generics and originator medicines in many product classes and disease areas, could help to improve perceptions of members of these stakeholder groups towards generic medicines. Furthermore, provision of the key information from such studies, in a non-technical, jargon-free and easy-to-understand manner to consumers, may have a positive impact on consumer/patient perception of generics. Continuation of such research in this field will ensure new and on-going information of equivalence (with some noted exceptions), safety and quality in the usage of generic medicines.

While many misconceptions are held within the patient group [69], a disturbing association can be observed in the literature regarding patient belief that generic and falsified (that is, counterfeit) medicines are the same [72, 15]. Given that generics are fully authorised, off-patent versions of branded medications, and very different from falsified medicines, this indicates that any educational interventions focused on this cohort need to include at least some information that explains the difference between generic and falsified medicines, in order to remove this falsely held belief as a source of mistrust of generics.

As several publications report that a previous positive experience with a generic medicine is more likely to improve patients’ positive opinions of, and confidence in, generic medicines [15, 67, 69, 18] – combined with the influence and trust which patients demonstrate in physicians [17, 52] – it is arguable that if physicians (and pharmacists) spend time explaining the equivalence of generic medication to a patient at their first encounter, this will encourage use of the generics and, therefore, improve future use of generic medications by that consumer.

Increased usage of generics may be brought about through implementation of recommendations from a recent review, which suggested increases in International Nonproprietary Name (INN) prescribing (a benefit of which may be to improve patient familiarity with generic names of medicines and, thus, reduce reliance on brand names, in addition to reducing patient confusion when dispensed generic preparations of varying appearance/packaging on different occasions), financial incentives to support generic prescribing and prescribing restrictions or removal of products from reimbursement lists, amongst other suggestions [93].

### Limitations

This review was limited in that PubMed and Scopus were the only databases used for sourcing literature (however, PubMed and Scopus combined represent considerable coverage of reputable journals) and that articles not published in English were excluded from the scope of this review.

In this review, there was a balance achieved between the sensitivity of the searches (that is, the capture of as many relevant papers as possible) versus the specificity (whereby screening of very large numbers of papers would be required to identify those relevant to the focus of this review). These results, whereby PubMed proved more effective in identifying relevant publications than did Scopus, mirror results seen by Freeman et al. [94] who reported that PubMed proved more specific than Google Scholar in locating relevant primary literature when comparing effective search databases for drug-related topics. Furthermore, Falagas et al. [95] reported that the keyword search with PubMed offers optimal update frequency and includes online early articles, although Scopus offers more coverage of journals. PubMed, however, remained an optimal tool in biomedical electronic research [95].

For qualitative reports, the impact of the intricacies of the relevant social contexts and methodologies (participant observation, ethnography, interviews, focus groups, textural or conversational) were not delved into beyond determining whether represented biases were detrimental to the findings of the studies.

Recognising that there has been a historical difficulty in including qualitative research easily into systematic review methodologies [96], considerable emphasis has been placed on assessing each of the reports included here using criteria for assessment of qualitative research promoted by the *British Medical Journal* [97] and Letts et al. [98]. In doing so, instead of detailing each aspect of each paper, the focus has been placed on the believability, robustness and transferability of the studies. Beyond that, therefore, there is a presumed credibility with respect to the researchers involved, reinforced to a great extent by the cumulative validation attributable due to the clear alignment of individual studies with one another and the triangulation with data presented in the quantitative papers.

With respect to quantitative studies, the question of whether the cohorts of subjects were (statistically or demographically) representative of their target communities was critiqued. Where elements of any paper were found to be weak in this regard, this has been detailed in the text above.

## Conclusion

While acceptance of generic medications is improving, substantial mistrust and lack of confidence remains, particularly within the patient and, to a lesser extent, physician groups. A key factor in improving the confidence of these cohorts is the provision of information and education, particularly in the areas of equivalency, regulation and in dispelling myths about generic medicines (such as the belief that they are counterfeits). Moreover, as patient trust in their physician often overrules their personal mistrust of generic medicines, improving the opinions of generics within the physician cohort may be of critical importance to improve usage and acceptance of generic medicines in the future. Given that reports indicate that patients who have a positive initial experience with a generic are more likely to maintain a positive opinion into the future, the physician–patient relationship and interaction may be key to influencing improving patient approval of generic medicines. To substantiate this facet of generic medicines, it may be useful for all stakeholders were a Cochrane review to be completed in this area; as of July 2014, a search of the Cochrane Database of Systematic Reviews found that no Cochrane Review had been published and no protocol title registered focused on patient, pharmacist and physician perspectives on generic medicines (with reference to Cochrane’s Primary Health Care, Health Care of Older People and Effective Practice and Organisation of Care groups).

## Abbreviations

AED: Antiepileptic drug
AUC: Area under the curve
CASP: Critical Appraisal Skills Programme
Cmax: Mean peak serum drug concentration
EMA: European Medicines Agency
FDA: US Food and Drug Administration
GP: General practitioner
GS: Generic substitution
INN: International Nonproprietary Name
MeSH: Medical Subject Headings
OTC: Over-the-counter
RTC: randomised controlled trial
WHO: World Health Organization
